# Supplementary material for: Enhancing CAR-T cell functionality in a patient-specific manner
Source: Nat Commun. 2023 Jan 31;14:506. doi: 10.1038/s41467-023-36126-7 (PMC9889707; doi:10.1038/s41467-023-36126-7)
Supplement: Supplementary file 1 — Supplementary Information [file 41467_2023_36126_MOESM1_ESM.pdf]

Supplementary Information

Enhancing CAR-T cell functionality in a patient-specific manner

David KY Zhang[1,2], Kwasi Adu-Berchie[1,2§], Siddharth Iyer[1,2§], Yutong Liu[1,2], Nicoletta Cieri[4], Joshua M Brockman[1,2], Donna Neuberg[5], Catherine J Wu[4,6,7,8], David J Mooney[1,2]

[1]John A. Paulson School of Engineering and Applied Sciences, Harvard University, Cambridge, Massachusetts, USA. [2]The Wyss Institute for Biologically Inspired Engineering, Harvard University, Cambridge, Massachusetts, USA. [4]Department of Medical Oncology. Dana-Farber Cancer Institute. [5]Department of Data Science, Dana-Farber Cancer Institute. [6]Department of Medicine, Brigham and Women's Hospital. [7]Harvard Medical School. [8]Broad Institute of MIT and Harvard.

Correspondence: David J Mooney (mooneyd@seas.harvard.edu)

Supplementary Figures

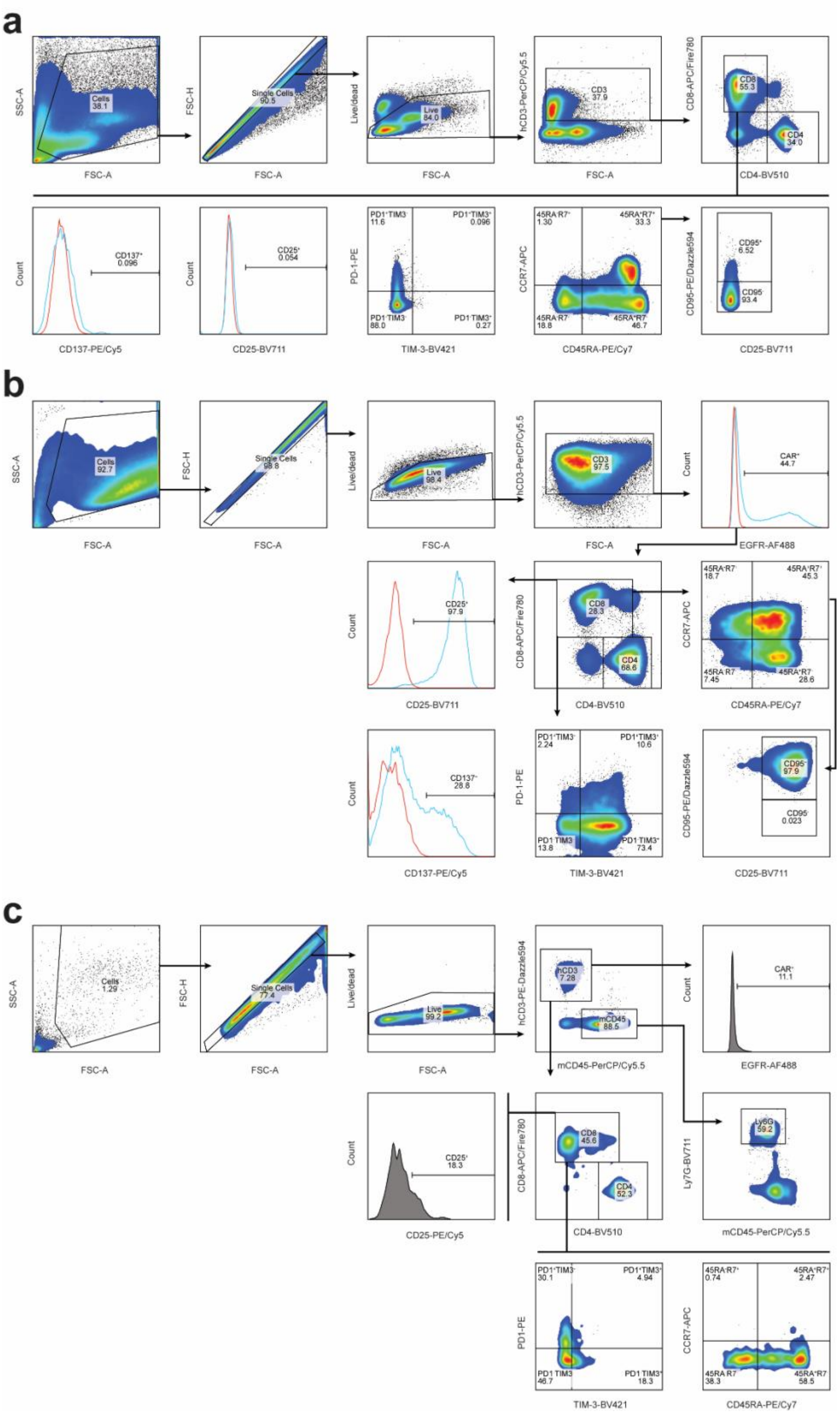

Supplementary Fig 1. Gating strategies employed in study. Sample gating strategy for T-cell blood samples (a), CAR-T cell products (b), and T cells harvested from animal blood in xenograft lymphoma model studies (c). Figs. 1a-c and associated Supplementary Figures used the gating strategy outlined in (a), while Fig. 1g-i used the gating strategy outlined in (b). The studies in Fig. 3 and related Supplementary Figures used the gating strategy outlined in (c). Mean fluorescence intensities (MFI) used in PCA analysis of T-cell blood samples included: MFI of CD3-PerCP/Cy5.5, CD4-BV510, CD8-APC/Fire750, PD-1-PE, Tim-3-BV421, CD45RA-PE/Cy7, CCR7-APC, CD95-PE/Dazzle594, CD25-BV711, CD137-PE/Cy5. Similarly, MFI and T-cell product features used in PCA analysis of CAR-T cell products included: T-cell fold expansion, stimulation dose (pg/cell), CD3-PerCP/Cy5.5, CD4-BV510, CD8-APC/Fire750, PD-1-PE, Tim-3-BV421, CD45RA-PE/Cy7, CCR7-APC, CD95-PE/Dazzle594, CD25-BV711, CD137-PE/Cy5, and EGFR-AF488 (as a proxy for CAR-expression). Gated frequencies and T-cell features used in analysis of CAR-T cell products included: Day 8 T-cell fold expansion, Day 8 CD4:CD8 ratio, %EGFR$^+$ of CD3$^+$ T cells, %CD45RA$^+$CCR7$^+$ of CD4$^+$ or CD8$^+$ T cells, %CD45RA$^+$CCR7$^-$ of CD4$^+$ or CD8$^+$ T cells, %CD45RA$^-$CCR7$^+$ of CD4$^+$ or CD8$^+$ T cells, %CD45RA$^-$CCR7$^-$ of CD4$^+$ or CD8$^+$ T cells, %PD-1$^+$Tim-3$^+$ of CD4$^+$ or CD8$^+$ T cells, %PD-1$^+$Tim-3$^-$ of CD4$^+$ or CD8$^+$ T cells, %PD-1$^-$Tim-3$^+$ of CD4$^+$ or CD8$^+$ T cells, %PD-1$^-$Tim-3$^-$ of CD4$^+$ or CD8$^+$ T cells, %CD25$^+$ of CD4$^+$ or CD8$^+$ T cells, %CD137$^+$ of CD4$^+$ or CD8$^+$ T cells. %CAR$^+$ cells were identified via truncated EGFR (tEGFR) staining. Red lines represent fluorescence-minus-one (FMO) controls.

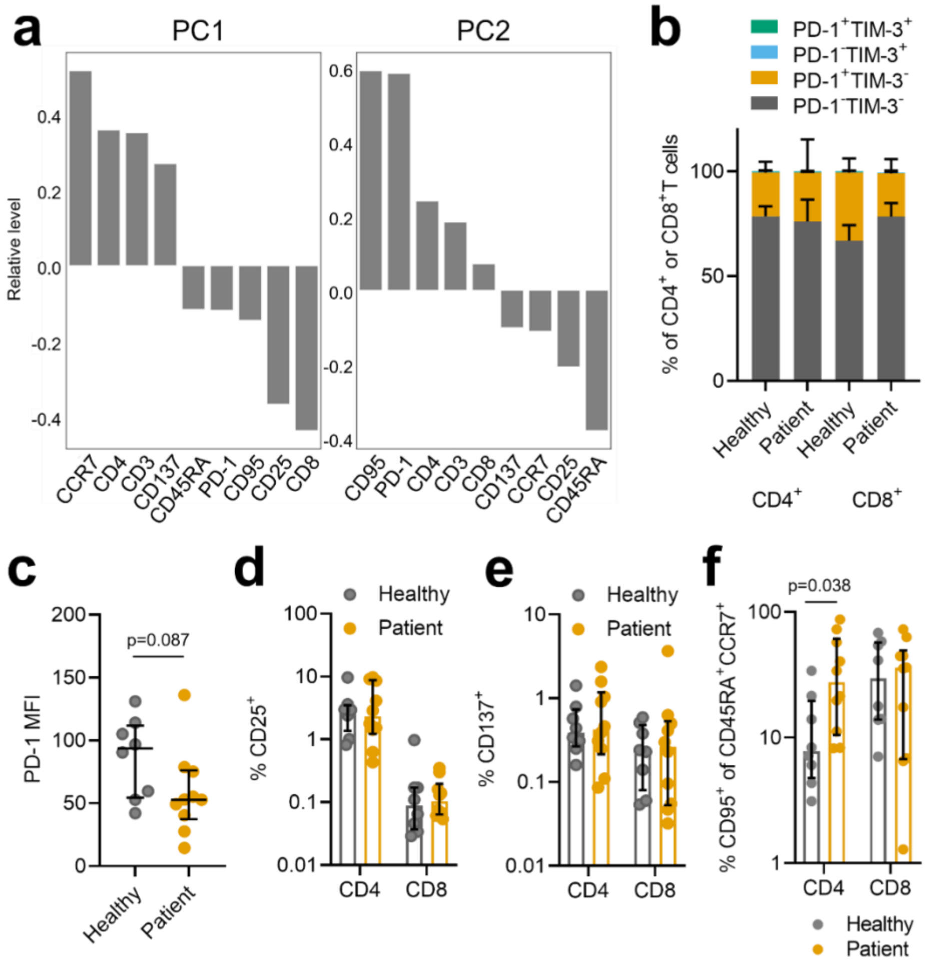

Supplementary Fig 2. Additional characterization of apheresis samples. (a) Contribution of various T-cell features to principal component 1 and principal component 2 in T-cell blood samples. (b) Expression of PD-1 and TIM-3 subpopulations among $CD4^+$ or $CD8^+$ T cells in healthy or patient-derived contexts. (c) Mean fluorescence intensity of PD-1 expression in healthy donor vs patient-derived T cells. Expression of CD25 (d) or CD137 (e) among healthy donor or patient-derived $CD4^+$ or $CD8^+$ T cells. (f) Frequency of $CD95^+$ $CD45RA^+CCR7^+$ CD4 and CD8 T cells in healthy or patient-derived blood samples. Data represents median ± interquartile range. n=8 for healthy donor T cells. n=10 for patient sample T cells. Comparisons in (c) and (f) calculated using two-sided Mann-Whitney tests.

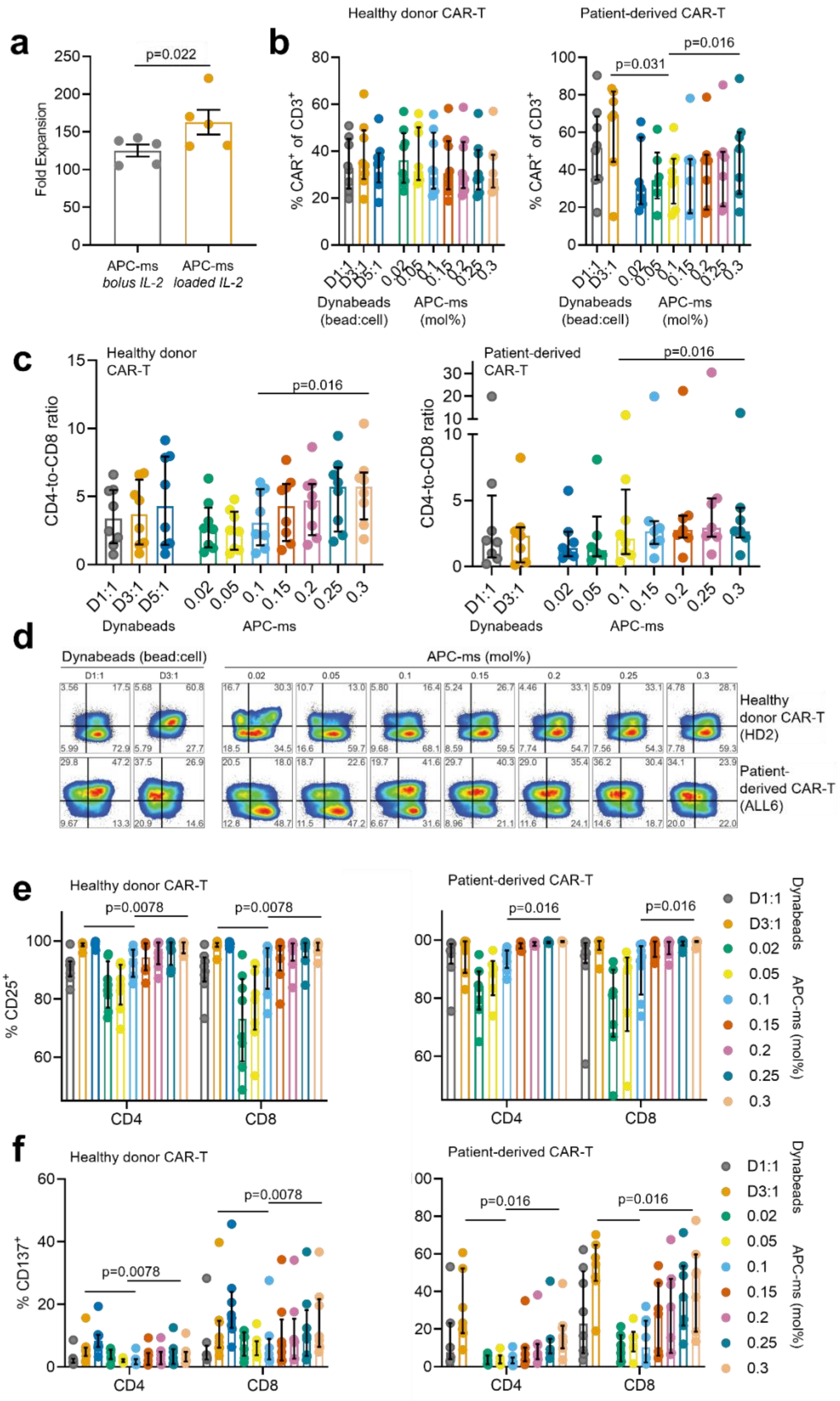

a
p=0.022
Fold Expansion
APC-ms bolus IL-2
APC-ms loaded IL-2
b
Healthy donor CAR-T
Patient-derived CAR-T
p=0.031
p=0.016
% CAR+ of CD3+
Dynabeads (bead:cell)
APC-ms (mol%)
c
Healthy donor CAR-T
Patient-derived CAR-T
p=0.016
CD4-to-CD8 ratio
Dynabeads
APC-ms
d
Dynabeads (bead:cell)
APC-ms (mol%)
Healthy donor CAR-T (HD2)
Patient-derived CAR-T (ALL6)
e
Healthy donor CAR-T
Patient-derived CAR-T
p=0.0078
p=0.016
% CD25+
CD4
CD8
D1:1
D3:1
0.02
0.05
0.1
0.15
0.2
0.25
0.3
Dynabeads
APC-ms (mol%)
f
Healthy donor CAR-T
Patient-derived CAR-T
p=0.0078
p=0.016
% CD137+
CD4
CD8

Supplementary Fig 3. Additional characterization of CAR-T cell products. (a) T-cell fold expansion in healthy donor T cells stimulated with APC-ms (0.1 mol%) without pre-loaded IL-2 (bolus) compared to pre-loaded IL-2 after 7 days. (b) CAR-transduction efficiency in healthy or patient-derived CAR-T cells across Dynabead and APC-ms stimulation conditions. (c) CD4:CD8 ratio in various healthy or patient-derived CAR-T cell products. (d) Representative FACS plots of $CD8^{+}$ CAR-T cells in various healthy or patient-derived CAR-T cell products. CD25 (e) and CD137 (f) in various healthy donor or patient-derived CAR-T cell products across Dynabead and APC-ms stimulation dose. Data in (a) represents n=5 healthy T-cell products and comparison was calculated using a paired student's T test. Data in (a) represents mean ± s.e.m. of n=5 healthy donor samples. Data in (b-c) and (e-f) represents median ± interquartile range. n=8 for healthy CAR-T cell products. n=8 for patient-derived CAR-T cell products. We were unable to manufacture CAR-T cells from one patient sample regardless of stimulation type, while only A0.1 and D1:1 groups were evaluated for a separate patient sample due to limited starting material. Comparisons in (b-c) and (e-f) calculated using two-sided Wilcoxon signed rank tests. Comparisons between healthy donor and patient-derived CAR-T cell products were calculated using two-sided Mann-Whitney tests.

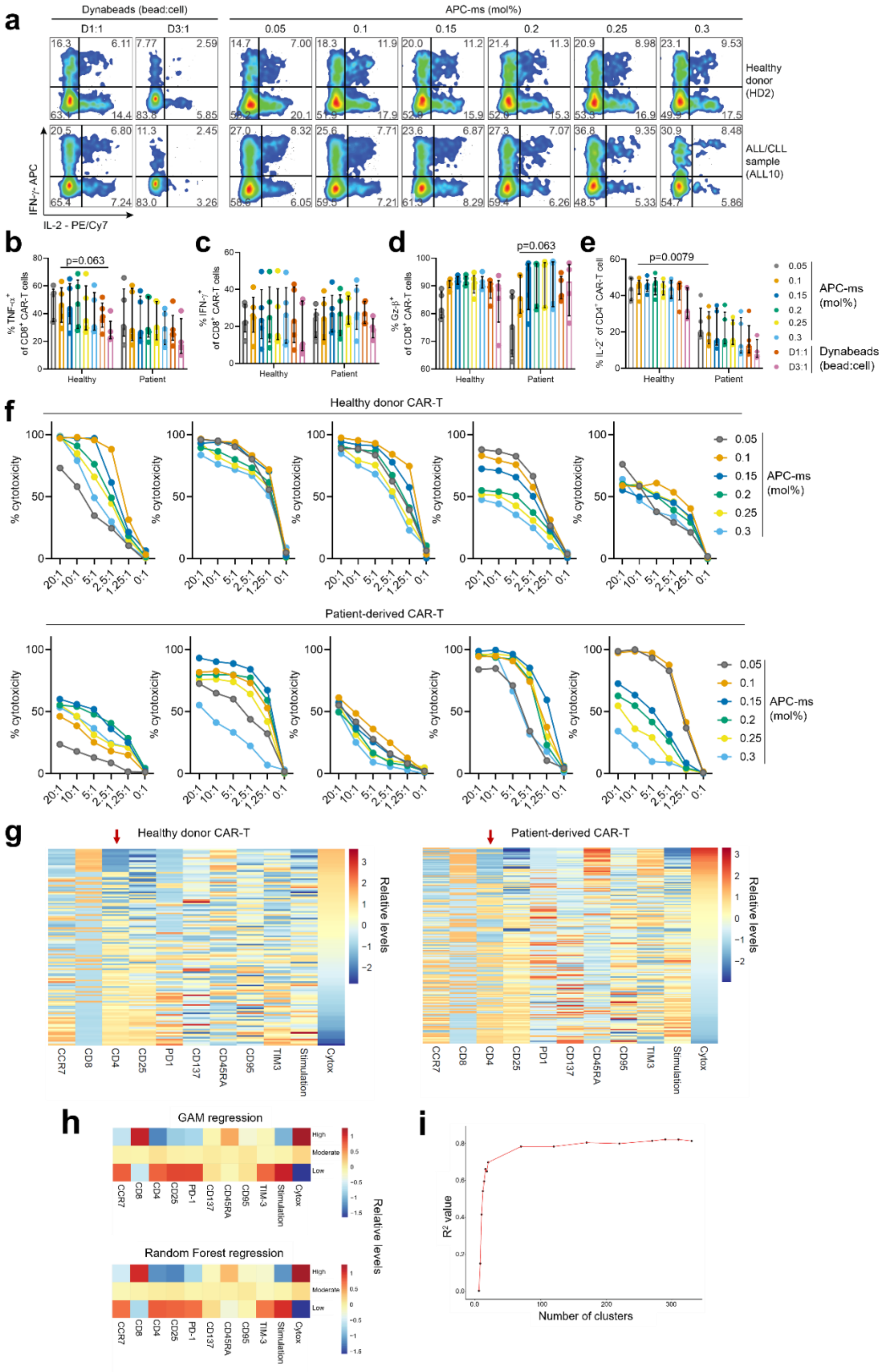

a
Dynabeads (bead:cell)
APC-ms (mol%)
D1:1
D3:1
0.05
0.1
0.15
0.2
0.25
0.3
Healthy donor (HD2)
ALL/CLL sample (ALL10)
IFN-γ - APC
IL-2 - PE/Cy7
b
p=0.063
Healthy
Patient
c
d
p=0.063
e
p=0.0079
APC-ms (mol%)
Dynabeads (bead:cell)
f
Healthy donor CAR-T
Patient-derived CAR-T
% cytotoxicity
g
Healthy donor CAR-T
Patient-derived CAR-T
Relative levels
CCR7
CD8
CD4
CD25
PD1
CD137
CD45RA
CD95
TIM3
Stimulation
Cytox
h
GAM regression
Random Forest regression
High
Moderate
Low
i
R² value
Number of clusters

Supplementary Fig 4. Supplemental characterization of CAR-T cell function. (a) Representative FACS plots of IFN-γ and IL-2 expression in $CD8^+$ CAR-T cells across a range of effector to target (E:T) ratios following co-culture with Raji-luc cells. Expression of TNF-α (b), IFN-γ (c), and granzyme-β (d) in various healthy donor or patient-derived CAR-T cell products following Raji-cell co-culture. (e) Expression of IL-2 in $CD4^+$ CAR-T cells following Raji-cell co-culture. (f) Cytotoxicity curves of representative healthy (top) and patient-derived (bottom) CAR-T cell samples across various APC-ms stimulation dose and at various E:T ratios. CAR-T cell products were phenotypically classified based on cytotoxic capacity using *K*-means clustering. (g) Heatmaps showing phenotype of various CAR-T cell clusters ordered by cytotoxic potential among healthy (left) or patient (right) CAR-T cell products. The red arrow indicated that $CD4^+$ T cells were not classified as being highly cytotoxic. An effector:target (E:T) ratio of 1.25:1 was used to generate the data in (b-d). (h) Comparison of GAM and Random Forest Regression: Heatmaps $CD8^+$ CAR-T cell markers associated with either high, moderate, or low levels of cytotoxicity. (i) R^2 value as a function of increasing k (to select the optimal k for K-means clustering and downstream GAM regression). Data in (b-e) represents median ± interquartile range. n=5 for healthy donor CAR-T cells. n=5 for patient-derived CAR-T cells. For the D3:1 group, n=4 patient samples were included. Comparisons in (b-e) calculated using two-sided Wilcoxon signed rank tests. Comparisons between healthy donor and patient-derived CAR-T cell products were calculated using two-sided Mann-Whitney tests.

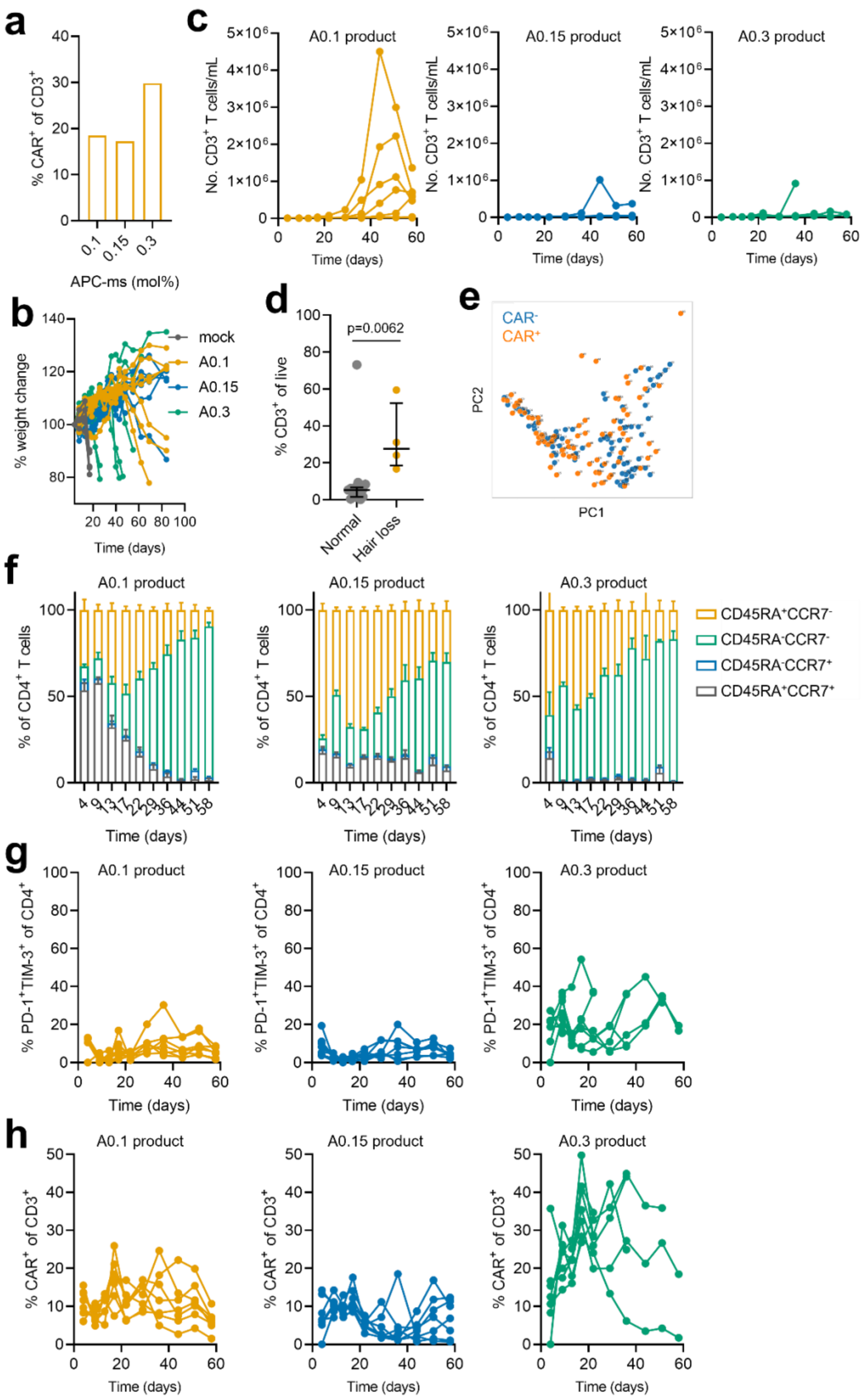
a
c
b
d
e
f
g
h
% CAR+ of CD3+
APC-ms (mol%)
A0.1 product
A0.15 product
A0.3 product
No. CD3+ T cells/mL
Time (days)
% weight change
mock
A0.1
A0.15
A0.3
% CD3+ of live
p=0.0062
Normal
Hair loss
CAR-
CAR+
PC1
PC2
% of CD4+ T cells
CD45RA+CCR7-
CD45RA-CCR7-
CD45RA-CCR7+
CD45RA+CCR7+
% PD-1+TIM-3+ of CD4+

Supplementary Fig 5. Additional phenotyping of T cells *in vivo*. (a) CAR transduction efficiency of A0.1, A0.15, and A0.3 CAR-T cell products. (b) Weight change in animals following adoptive transfer of A0.1, A0.15, and A0.3. (c) Concentration of total CD3$^+$ T cells in circulation following dosing, highlighting expansive *in vivo* proliferation in the A0.1 product. (d) Frequency of CD3$^+$ T cells in animals with and without observable hair loss. (e) Principal component analysis of CAR$^+$ and CAR$^-$ T cells. The PCA represents the phenotype of harvested T cells from A0.1, A0.15, and A0.3 treated mice consolidated across four timepoints (day 9, 13, 17, and 22). No significant differences were observed between CAR$^+$ and CAR$^-$ cell phenotype. Frequency of various CD45RA and CCR7-expressing CD4$^+$ subpopulations (f), PD-1 and TIM-3 co-expression among CD4$^+$ T cells (g), and CAR-expression (h) among harvested T cells from A0.1, A0.15, and A0.3 product-treated mice over time. Data represent CAR-T cell products generated via APC-ms from patient-sample ALL10: mock, n=5; A0.1 (0.1 mol% APC-ms stimulated CAR-T cells), n=7; A0.15 (0.15 mol% APC-ms stimulated CAR-T cells), n=7; A0.3 (0.3 mol% APC-ms stimulated CAR-T cells), n=7 mice. Comparison in (d) determined using a two-sided Mann-Whitney test. Data in (d) represents median ± interquartile range. Data in (f) represents mean ± s.e.m.

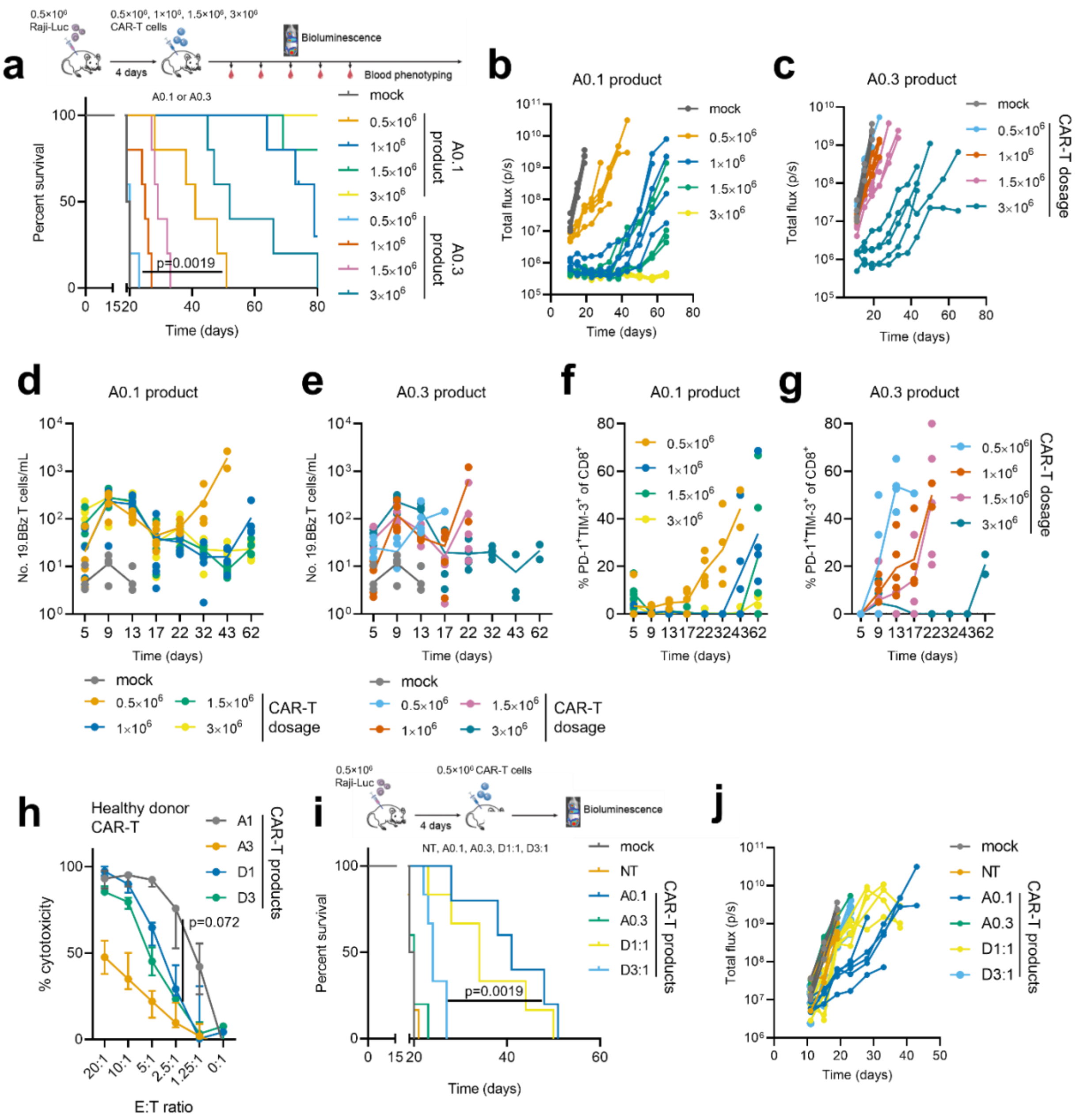

Supplementary Fig 6. Evaluation of the stimulation dose dependency of CAR-T cell responsiveness in a healthy donor sample. (a) Study outline of disseminated Raji xenograft model and animal survival. $5{\times}10^{\mathbf{5}}$ luciferized Raji cells were administered to NSG mice four days prior to treatment with $0.5{\times}10^6$, $1{\times}10^6$, $1.5{\times}10^6$, or $3{\times}10^6$ CAR$^+$ T cells. CAR-T cells were generated from healthy donor sample HD1 using APC-ms presenting 0.1 mol% or 0.3 mol% anti-CD3/anti-CD28 polyclonal stimulation. mock, n=4; A0.1 (0.1 mol% APC-ms stimulated CAR-T cells), n=5; A0.3 (0.3 mol% APC-ms stimulated CAR-T cells), n=5 mice. One animal in the A0.1 product, $1{\times}10^6$ CAR-T cell dosage group was excluded due to non-tumor related complications. Quantification of bioluminescent signal following infusion with A0.1 product (b) and the A0.3 product (c). (d-e) *In vivo* concentration of circulating CAR-T cells following infusion. Change in PD-1/Tim-3 co-expression in CD8$^+$ T cells in animals treated with the A0.1 product (f) or the A0.3 product (g). (h) *In vitro* cytotoxic potential of CAR-T cell products generated from either APC-ms presenting 0.1 or 0.3 mol % anti-CD3/anti-CD28 (A0.1, A0.3 products respectively) or Dynabeads

dosed at either a 1:1 or 3:1 bead:cell ratio (D1:1, D3:1 products respectively). (i) Study outline of disseminated Raji xenograft model and animal survival. 5×10^{5} luciferized Raji cells were administered to NSG mice four days prior to treatment with 0.5×10^{6} A0.1, A0.3, D1:1 or D3:1 CAR-T cells. NT represents non-transduced APC-ms-primed CAR^{-} T cells, n=6. 5×10^{6} NT cells were infused. (j) Quantification of bioluminescent signal. Data in (h) represents median ± interquartile range of n=5 healthy CAR-T cell samples. The comparison in (h) was calculated using a two-sided Wilcoxon signed-rank test. Animal survival in (a) and (i) was calculated using a two-tailed log-rank Mantel-Cox test.

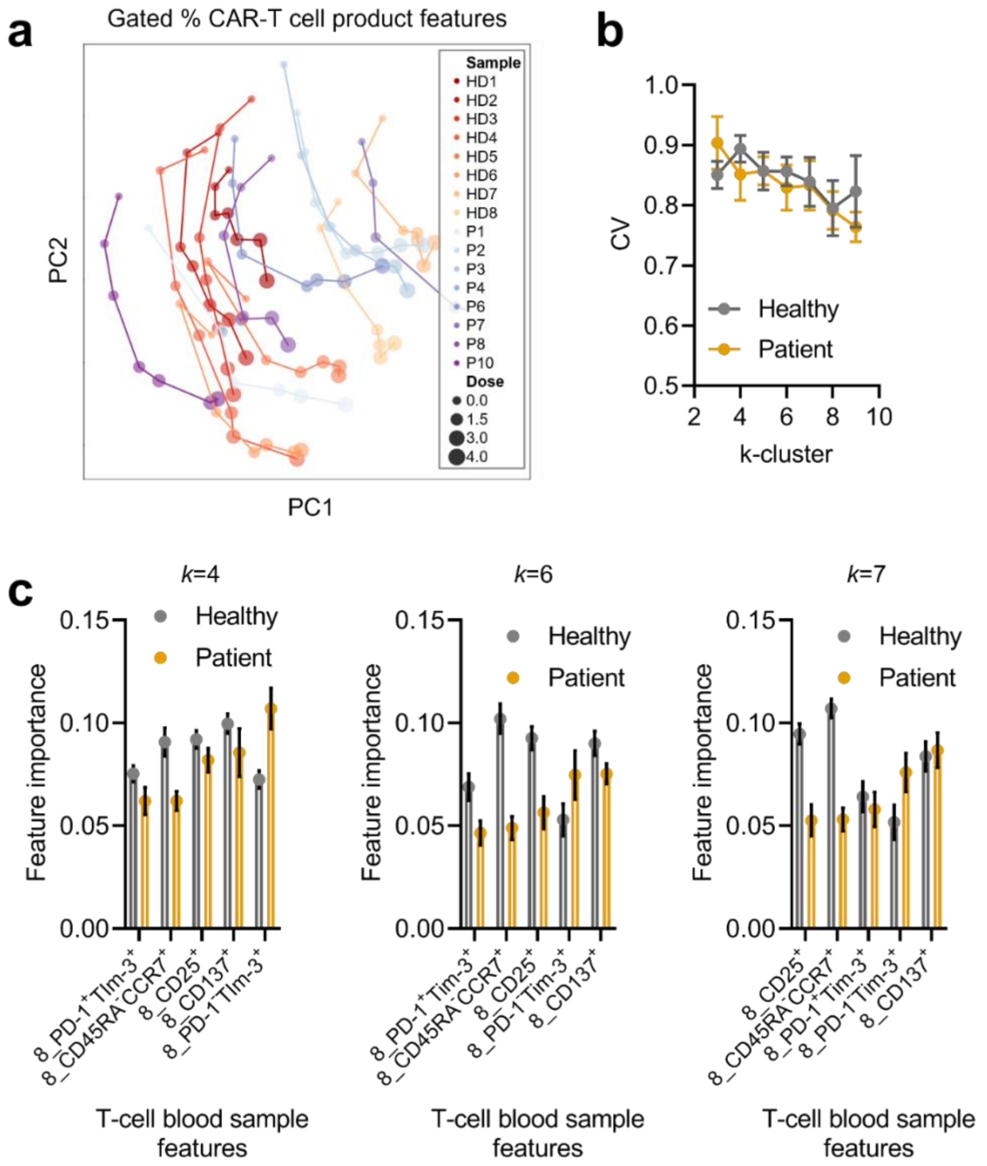

Supplementary Fig 7. Modeling blood samples and CAR-T cell products. (a) PCA of gated CAR-T cell frequencies as features, colored by healthy donor or patient-derived sample. The size of the circle represents different APC-ms stimulation doses in log(pg/cell) and the lines connect CAR-T cell products derived from the same healthy or patient sample. HD: healthy donor; P: patient. A standard 12-marker FACS panel and gating strategy were used (Supplementary Fig. 1b). (b) 10-fold cross validation scores with increasing *k*. *K*-means clustering was performed on the data in (a) separately for healthy and patient CAR-T cell samples. *k=5* was selected for downstream classification modeling. Ten iterations were used to calculate the mean and variance. (c) Top five most important features in the classification model importance at *k*=4,6, and 7, following stimulation dose*.* All important features were from $CD8^+$ T cells (as indicated by "8_") in the blood sample. Data in (b-c) represents mean ± s.d.

Tables

Supplementary Table 1. Treatment history and status at time of sampling of ALL and CLL patient samples

Sample ID	Last therapy (TX)	Time from last TX to sampling	Type of therapy
ALL1	chemo	NA (≤1 month)	HyperCVAD
CLL2	chemo	NA (≥1 month)	R-DA-EPOCH
CLL3	chemo	NA (≥1 month)	R-Bendamustine
ALL4	chemo	ongoing at sampling	MTX-6MP maintenance
CLL5	chemo	4 months	FCR
ALL6	chemo	ongoing at sampling	Larson regimen early intensification
ALL7	immunotherapy	NA (≥1 month)	Inotuzumab
CLL8	chemo	NA (≥1 month)	R-CHOP $^{+}$ 3 HDMTX
CLL9	BTK inhibitor	ongoing at sampling	Ibrutinib
ALL10	chemo	ongoing at sampling	6MP

Supplementary Table 2. MSR characterization

Physical dimensions*	Aspect ratio	Surface area* (m^2/g)	Pore volume* (cm^3/g)	Pore radius* (nm)
Length: 83.0 ± 32.3 μm<br>(range:18.9 - 213.2 μm)<br>Width: 14.6 ± 3.3 μm<br>(range: 4.5 - 23.0 μm)	~6	570 ± 70	0.64 ± 0.07	32 ± 0.64

*Physical dimensions were measured using ImageJ. Surface area was calculated from nitrogen sorption isotherms using the BET method (using 77K, and isotherm adsorption data with P/P0 from 0.05 to 0.3). The pore diameter and volume were calculated with an adsorption branch using the BJH method. Data represents mean ± S.D.

Supplementary Table 3. APC-ms formulations used in study

APC-ms formulation	Fraction of lipid presenting activating stimulus (mol %)	Activating αCD3/αCD28 stimulus (ng/ug APC-ms)	Stimulation dose (pg 1:1 αCD3/αCD28 /cell)
A0.02	0.02	0.8	0.24
A0.05	0.05	2	0.6
A0.1	0.1	4	1.2
A0.15	0.15	6	1.8
A0.2	0.2	8	2.4
A0.25	0.25	10	3.0
A0.3	0.3	12	3.6

Supplementary Table 4. Cross-validated prediction scores of various classification models with and without the inclusion of T-cell sample features

With Features	Model type	Random forest	MLPC	Decision Tree	Nearest Neighbors	Naïve Bayes
	Healthy	0.82 ± 0.09	0.80 ± 0.06	0.78 ± 0.14	0.59 ± 0.12	0.60 ± 0.17
	ALL/CLL	0.73 ± 0.10	0.62 ± 0.10	0.63 ± 0.12	0.46 ± 0.17	0.53 ± 0.12
Only Stimulation	Model type	Random forest	MLPC	Decision Tree	Nearest Neighbors	Naïve Bayes
	Healthy	0.29 ± 0.09	0.37 ± 0.12	0.29 ± 0.10	0.30 ± 0.10	0.41 ± 0.09
	ALL/CLL	0.29 ± 0.10	0.34 ± 0.07	0.25 ± 0.12	0.23 ± 0.09	0.38 ± 0.10

Supplementary Table 5. Detailed statistical analyses and p-values.

Figure	Comparison	Analysis	P-value
Fig. 1c	Healthy vs patient CD4:CD8	Mann-Whitney test	*p=0.0062
Fig. 1d	Healthy vs patient $CD8^{+}$ $CD45RA^{+}CCR7^{-}$	Mann-Whitney test	*p=0.0062
Supplementary Fig. 2c	Healthy vs patient $CD3^{+}$PD-1 MFI	Mann-Whitney test	p=0.087
Supplementary Fig. 2f	Healthy vs patient $CD4^{+}$ $CD45RA^{+}CCR7^{+}CD95^{+}$	Mann-Whitney test	*p=0.038
Fig. 1g	Healthy vs patient A0.1 fold expansion	Mann-Whitney test	*p=0.05
Fig. 1g	Healthy A0.1 vs A0.3 fold expansion	Wilcoxon signed rank test	p=0.74
Fig. 1g	Healthy A0.1 vs D3:1 fold expansion	Wilcoxon signed rank test	p=0.38
Fig. 1g	Patient A0.1 vs A0.3 fold expansion	Wilcoxon signed rank test	p=0.078
Fig. 1g	Patient A0.1 vs D3:1 fold expansion	Wilcoxon signed rank test	p=0.11
Supplementary Fig. 3a	APC-ms IL-2 bolus vs APC-ms/IL-2 loaded	Paired student's T test	*p=0.022
Supplementary Fig. 3b	Healthy vs Patient A0.1 CAR^{+}	Mann-Whitney test	p=0.80
Supplementary Fig. 3b	Healthy A0.1 vs A0.3 CAR^{+}	Wilcoxon signed rank test	p=0.31
Supplementary Fig. 3b	Healthy A0.1 vs D3:1 CAR^{+}	Wilcoxon signed rank test	p=0.15
Supplementary Fig. 3b	Patient A0.1 vs A0.3 CAR^{+}	Wilcoxon signed rank test	*p=0.016
Supplementary Fig. 3b	Patient A0.1 vs D3:1 CAR^{+}	Wilcoxon signed rank test	*p=0.031
Fig. 1h	Healthy $CD4^{+}$ A0.1 vs A0.3 PD-1 and Tim-3 co-expression	Wilcoxon signed rank test	*p=0.0078
Fig. 1h	Healthy $CD8^{+}$ A0.1 vs A0.3 PD-1 and Tim-3 co-expression	Wilcoxon signed rank test	*p=0.016
Fig. 1h	Healthy $CD4^{+}$ A0.1 vs D3:1 PD-1 and Tim-3 co-expression	Wilcoxon signed rank test	p=0.0547
Fig. 1h	Healthy $CD8^{+}$ A0.1 vs D3:1 PD-1 and Tim-3 co-expression	Wilcoxon signed rank test	p=0.31
Fig. 1h	Patient $CD4^{+}$ A0.1 vs A0.3 PD-1 and Tim-3 co-expression	Wilcoxon signed rank test	*p=0.016
Fig. 1h	Patient $CD8^{+}$ A0.1 vs A0.3 PD-1 and Tim-3 co-expression	Wilcoxon signed rank test	*p=0.016
Fig. 1h	Patient $CD4^{+}$ A0.1 vs D3:1 PD-1 and Tim-3 co-expression	Wilcoxon signed rank test	*p=0.016
Fig. 1h	Patient $CD8^{+}$ A0.1 vs D3:1 PD-1 and Tim-3 co-expression	Wilcoxon signed rank test	*p=0.031
Supplementary Fig. 3c	Healthy A0.1 vs A0.3 CD4:CD8	Wilcoxon signed rank test	*p=0.016
Supplementary Fig. 3c	Healthy A0.1 vs D3:1 CD4:CD8	Wilcoxon signed rank test	p=0.31
Supplementary Fig. 3c	Patient A0.1 vs A0.3 CD4:CD8	Wilcoxon signed rank test	*p=0.016
Supplementary Fig. 3c	Patient A0.1 vs D3:1 CD4:CD8	Wilcoxon signed rank test	p=0.47
Fig. 1i	Healthy A0.15 vs A0.3 $CD45RA^{+}CCR7^{+}$ $CD8^{+}CAR^{+}$	Wilcoxon signed rank test	p=0.20
Fig. 1i	Healthy A0.15 vs A0.3 $CD45RA^{+}CCR7^{-}$ $CD8^{+}CAR^{+}$	Wilcoxon signed rank test	p=0.84
Fig. 1i	Patient A0.15 vs A0.3 $CD45RA^{+}CCR7^{+}$ $CD8^{+}CAR^{+}$	Wilcoxon signed rank test	*p=0.016
Fig. 1i	Patient A0.15 vs A0.3 $CD45RA^{+}CCR7^{-}$ $CD8^{+}CAR^{+}$	Wilcoxon signed rank test	p=0.078
Supplementary Fig. 3e	Healthy A0.1 vs A0.3 $CD4^{+}$ CD25	Wilcoxon signed rank test	*p=0.0078
Supplementary Fig. 3e	Healthy A0.1 vs D3:1 $CD4^{+}$ CD25	Wilcoxon signed rank test	*p=0.0078

Supplementary Fig. 3e	Patient A0.1 vs A0.3 $CD4^+$ CD25	Wilcoxon signed rank test	p=0.156
Supplementary Fig. 3e	Patient A0.1 vs D3:1 $CD4^+$ CD25	Wilcoxon signed rank test	p=0.67
Supplementary Fig. 3e	Healthy A0.1 vs A0.3 $CD8^+$ CD25	Wilcoxon signed rank test	*p=0.0078
Supplementary Fig. 3e	Healthy A0.1 vs D3:1 $CD8^+$ CD25	Wilcoxon signed rank test	*p=0.0078
Supplementary Fig. 3e	Patient A0.1 vs A0.3 $CD8^+$ CD25	Wilcoxon signed rank test	p=0.156
Supplementary Fig. 3e	Patient A0.1 vs D3:1 $CD8^+$ CD25	Wilcoxon signed rank test	p=0.469
Supplementary Fig. 3f	Healthy A0.1 vs A0.3 $CD4^+$ CD137	Wilcoxon signed rank test	*p=0.0078
Supplementary Fig. 3f	Healthy A0.1 vs D3:1 $CD4^+$ CD137	Wilcoxon signed rank test	*p=0.0078
Supplementary Fig. 3f	Patient A0.1 vs A0.3 $CD4^+$ CD137	Wilcoxon signed rank test	p=0.156
Supplementary Fig. 3f	Patient A0.1 vs D3:1 $CD4^+$ CD137	Wilcoxon signed rank test	p=0.156
Supplementary Fig. 3f	Healthy A0.1 vs A0.3 $CD8^+$ CD137	Wilcoxon signed rank test	*p=0.0078
Supplementary Fig. 3f	Healthy A0.1 vs D3:1 $CD8^+$ CD137	Wilcoxon signed rank test	*p=0.0078
Supplementary Fig. 3f	Patient A0.1 vs A0.3 $CD8^+$ CD137	Wilcoxon signed rank test	p=0.156
Supplementary Fig. 3f	Patient A0.1 vs D3:1 $CD8^+$ CD137	Wilcoxon signed rank test	p=0.156
Fig. 2b	Healthy vs Patient A0.1 $CD8^+$ CAR-T cells $IL\text{-}2^+$	Mann-Whitney test	*p=0.0079
Fig. 2b	Healthy A0.1 vs A0.3 $CD8^+$ $IL\text{-}2^+$	Wilcoxon signed rank test	p=0.19
Fig. 2b	Healthy A0.1 vs D3:1 $CD8^+$ $IL\text{-}2^+$	Wilcoxon signed rank test	p=0.063
Fig. 2b	Patient A0.1 vs A0.3 $CD8^+$ $IL\text{-}2^+$	Wilcoxon signed rank test	p=0.25
Fig. 2b	Patient A0.1 vs D3:1 $CD8^+$ $IL\text{-}2^+$	Wilcoxon signed rank test	p=0.13
Supplementary Fig. 4b	Healthy vs Patient A0.1-D3:1 $CD8^+$ CAR-T cells $TNF\text{-}a^+$	Mann-Whitney test	p=0.42
Supplementary Fig. 4b	Healthy A0.1 vs A0.3 $CD8^+$ TNF-a $^+$	Wilcoxon signed rank test	p=0.44
Supplementary Fig. 4b	Healthy A0.1 vs D3:1 $CD8^+$ TNF-a $^+$	Wilcoxon signed rank test	p=0.063
Supplementary Fig. 4b	Patient A0.1 vs A0.3 $CD8^+$ TNF-a $^+$	Wilcoxon signed rank test	p=0.19
Supplementary Fig. 4b	Patient A0.1 vs D3:1 $CD8^+$ TNF-a $^+$	Wilcoxon signed rank test	p=0.13
Supplementary Fig. 4c	Healthy vs Patient A0.1-D3:1 $CD8^+$ CAR-T cells $IFN\text{-}g^+$	Mann-Whitney test	p=0.84
Supplementary Fig. 4c	Healthy A0.1 vs A0.3 $CD8^+$ IFN-g $^+$	Wilcoxon signed rank test	p=0.63
Supplementary Fig. 4c	Healthy A0.1 vs D3:1 $CD8^+$ IFN-g $^+$	Wilcoxon signed rank test	p=0.31
Supplementary Fig. 4c	Patient A0.1 vs A0.3 $CD8^+$ IFN-g $^+$	Wilcoxon signed rank test	p=0.63
Supplementary Fig. 4c	Patient A0.1 vs D3:1 $CD8^+$ IFN-g $^+$	Wilcoxon signed rank test	p=0.38
Supplementary Fig. 4d	Healthy vs Patient A0.1-D3:1 $CD8^+$ CAR-T cells $Gz\text{-}B^+$	Mann-Whitney test	p=0.65
Supplementary Fig. 4d	Healthy A0.1 vs A0.3 $CD8^+$ $Gz\text{-}B^+$	Wilcoxon signed rank test	p=0.63
Supplementary Fig. 4d	Healthy A0.1 vs D3:1 $CD8^+$ $Gz\text{-}B^+$	Wilcoxon signed rank test	p=0.31
Supplementary Fig. 4d	Patient A0.1 vs A0.3 $CD8^+$ $Gz\text{-}B^+$	Wilcoxon signed rank test	p=0.063
Supplementary Fig. 4d	Patient A0.1 vs D3:1 $CD8^+$ $Gz\text{-}B^+$	Wilcoxon signed rank test	p=0.88
Supplementary Fig. 4e	Healthy vs Patient A0.1 $CD4^+$ CAR-T cells $IL\text{-}2^+$	Mann-Whitney test	*p=0.0079
Fig. 2c	Healthy A0.1 vs A0.3 %cytotoxicity	Wilcoxon signed rank test	p=0.063
Fig. 2c	Healthy A0.1 vs D3:1 %cytotoxicity	Wilcoxon signed rank test	p=0.063
Fig. 2d	Patient A0.1 vs A0.3 %cytotoxicity	Wilcoxon signed rank test	p=0.063
Fig. 2d	Patient A0.1 vs D3:1 %cytotoxicity	Wilcoxon signed rank test	p=0.063
Fig. 3h	A0.1 or A0.15 vs A0.3 animal survival	Log-rank Mantel-Cox test	*p=0.003
Supplementary Fig. 5d	Normal vs Hair loss	Mann-Whitney test	*p=0.0062
Supplementary Fig. 6a	0.5×10^6 A0.1 vs 0.5×10^6 A0.3	Log-rank Mantel-Cox test	*p=0.0019
Supplementary Fig. 6h	A0.1 vs D3:1 %cytotoxicity	Wilcoxon signed rank test	p=0.072
Supplementary Fig. 6i	A0.1 vs D3:1 animal survival	Log-rank Mantel-Cox test	*p=0.0019

146
