# Supplementary material for: Enhancing CAR-T cell functionality in a patient-specific manner
Source: Nat Commun. 2023 Jan 31;14:506. doi: 10.1038/s41467-023-36126-7 (PMC9889707; doi:10.1038/s41467-023-36126-7)
Supplement: Supplementary file 3 — Reporting Summary [file 41467_2023_36126_MOESM3_ESM.pdf]

nature portfolio

Corresponding author(s): David J Mooney

Last updated by author(s): Dec 15, 2022

Reporting Summary

Nature Portfolio wishes to improve the reproducibility of the work that we publish. This form provides structure for consistency and transparency in reporting. For further information on Nature Portfolio policies, see our Editorial Policies and the Editorial Policy Checklist.

Statistics

For all statistical analyses, confirm that the following items are present in the figure legend, table legend, main text, or Methods section.

n/a	Confirmed	
☐	☒	The exact sample size (*n*) for each experimental group/condition, given as a discrete number and unit of measurement
☐	☒	A statement on whether measurements were taken from distinct samples or whether the same sample was measured repeatedly
☐	☒	The statistical test(s) used AND whether they are one- or two-sided<br>*Only common tests should be described solely by name; describe more complex techniques in the Methods section.*
☒	☐	A description of all covariates tested
☐	☒	A description of any assumptions or corrections, such as tests of normality and adjustment for multiple comparisons
☐	☒	A full description of the statistical parameters including central tendency (e.g. means) or other basic estimates (e.g. regression coefficient) AND variation (e.g. standard deviation) or associated estimates of uncertainty (e.g. confidence intervals)
☐	☒	For null hypothesis testing, the test statistic (e.g. *F*, *t*, *r*) with confidence intervals, effect sizes, degrees of freedom and *P* value noted<br>*Give P values as exact values whenever suitable.*
☒	☐	For Bayesian analysis, information on the choice of priors and Markov chain Monte Carlo settings
☒	☐	For hierarchical and complex designs, identification of the appropriate level for tests and full reporting of outcomes
☒	☐	Estimates of effect sizes (e.g. Cohen's *d*, Pearson's *r*), indicating how they were calculated

Our web collection on statistics for biologists contains articles on many of the points above.

Software and code

Policy information about availability of computer code

Data collection	Flow cytometry data was collected using a 5-laser BD Fortessa equipped with FACS Diva. No other software or custom code were used for data collection.
Data analysis	Statistical analysis was performed in Graphpad Prism v8.01 & v9.1.1, and R.<br>Data visualization and computational modeling was performed using Python and R. Custom code is available via Github at https://github.com/siddharthriyer/car_t_stimulation. Specific software packages for running the code is listed here: https://github.com/siddharthriyer/car_t_stimulation/blob/main/requirements.txt. Specifically, the following packages are required: numpy (1.21.5), pandas (1.4.1), matplotlib (3.5.1), seaborn (0.11.2), and scikit-learn (1.02)<br>Flow cytometry analysis was performed using FlowJo v10.8.1.<br>IVIS acquisition and analysis was performed in Living Image v5.

For manuscripts utilizing custom algorithms or software that are central to the research but not yet described in published literature, software must be made available to editors and reviewers. We strongly encourage code deposition in a community repository (e.g. GitHub). See the Nature Portfolio guidelines for submitting code & software for further information.

Data

Policy information about availability of data

All manuscripts must include a data availability statement. This statement should provide the following information, where applicable:

- Accession codes, unique identifiers, or web links for publicly available datasets
- A description of any restrictions on data availability
- For clinical datasets or third party data, please ensure that the statement adheres to our policy

All computational analyses and data used for model training have been made available at: https://github.com/siddharthriyer/car_t_stimulation. Patient-related data that was collected but not shown in the paper might be subject to confidentiality. All other relevant data requests should be made to the corresponding author.

Human research participants

Policy information about studies involving human research participants and Sex and Gender in Research.

Reporting on sex and gender	No human research participants were involved in this study. Patient samples were obtained from the Dana Farber CAncer Institute and included both sexes, but specific sex information is not available due to confidentiality.
Population characteristics	As the patient samples were obtained from separate clinical trials, population characteristics are not available.
Recruitment	As the patient samples were obtained from separate clinical trials, recruitment information is not available.
Ethics oversight	All handling of human patient samples were conducted in accordance to the Dana Farber Cancer Institute Institutional Review Board- approved protocols.

Note that full information on the approval of the study protocol must also be provided in the manuscript.

Field-specific reporting

Please select the one below that is the best fit for your research. If you are not sure, read the appropriate sections before making your selection.

☒ Life sciences ☐ Behavioural & social sciences ☐ Ecological, evolutionary & environmental sciences

For a reference copy of the document with all sections, see nature.com/documents/nr-reporting-summary-flat.pdf

Life sciences study design

All studies must disclose on these points even when the disclosure is negative.

Sample size	Sample sizes for animal studies were determined based on prior experience with the Xenograft models and G*Power a priori analysis (doi: 10.1038/nbt.4047, doi: 10.1073/pnas.1524155113). For experiments not-involving animals, no sample size calculation was performed. Approximately n=10 healthy donor or patient samples were chosen based on our past experience with inter-donor variability.
Data exclusions	Any animals that were excluded from the analyses are described in the figure captions. All exclusions were due to non-tumor related complications of the study, such as human error.
Replication	The experiments were repeated as described in the figure captions.
Randomization	Mice were randomized into the experimental groups following tumor inoculation prior to CAR-T cell dosing. For studies not involving animals, no randomization was required as each sample was either derived from a healthy donor or a patient, and was treated with all possible groups (e.g., APC-ms or Dynabeads). All computational datasets were split randomly.
Blinding	The investigators were not blinded in the animal studies when dosing the animals with CAR-T cells, as there is already inherent variability from injection to injection. Blinding was not relevant for studies not involving animals.

Reporting for specific materials, systems and methods

We require information from authors about some types of materials, experimental systems and methods used in many studies. Here, indicate whether each material, system or method listed is relevant to your study. If you are not sure if a list item applies to your research, read the appropriate section before selecting a response.

Materials & experimental systems

n/a	Involved in the study
☐	☒ Antibodies
☐	☒ Eukaryotic cell lines
☒	☐ Palaeontology and archaeology
☐	☒ Animals and other organisms
☒	☐ Clinical data
☒	☐ Dual use research of concern

Methods

n/a	Involved in the study
☒	☐ ChIP-seq
☐	☒ Flow cytometry
☒	☐ MRI-based neuroimaging

Antibodies

Antibodies used	Anti-human antibodies for flow cytometry were obtained from BioLegend: CD3-PerCP/Cy5.5 (HIT3a, cat. no. 300328), CD4-BV510 (SK3, cat. no. 344634), CD8-APC/Fire750 (SK1, cat. no. 344746), PD-1-PE (EH12.2H7, cat. no. 353410), TIM-3-BV421 (F38-2E2, cat. no. 345008), CD25-BV711 (M-A251, cat. no. 356138), CD45RA-PE/Cy7 (HI100, cat. no. 304126), CCR7-APC (G043H7, cat. no. 353214), truncated EGFR-AF488 (AY13, cat. no. 352908), CD95-PE/Dazzle (DX2, cat. no. 305634), CD137-PE/Cy5 (4B4-1, cat. no. 309808), CD3-PE/Dazzle (HIT3a, cat. no. 300336), CD25-PE/Cy5 (M-A251, cat. no. 356156), GranzymeB-BV421 (GB11, cat. no. 515408), IL-2-PE/Cy7 (MQ1-17H1, cat. no. 500326), IFNg-APC (4S.B3, cat. no. 502512), and TNFa-FITC (MAb11, cat. no. 502906). Anti-mouse CD45-PerCP/Cy5.5 (30-F11, cat. no. 103132) and Ly6G-BV711 (1A8, cat. no. 127643) were obtained from BioLegend. All antibodies were used at the manufacturer-recommended dilution. Biotinylated anti-CD3 (OKT3, cat. no. 317320) and anti-CD28 (CD28.2, cat. no. 302904) were obtained from Biolegend.
Validation	The antibodies used in this study have been validated by Biolegend.

Eukaryotic cell lines

Policy information about cell lines and Sex and Gender in Research

Cell line source(s)	Raji (CCL-86, ATCC), HEK293T (CRL-3216, ATCC)
Authentication	Authentication and associated testing was performed by ATCC. They confirmed that the Raji line tested positive for the presence of Epstein Barr virus (EBV) viral DNA sequences via PCR. STR profiling was performed by ATCC.
Mycoplasma contamination	Mycoplasma testing was performed by ATCC.
Commonly misidentified lines (See ICLAC register)	THP-1 (for Raji cells)

Animals and other research organisms

Policy information about studies involving animals; ARRIVE guidelines recommended for reporting animal research, and Sex and Gender in Research

Laboratory animals	Female NOD.Cg-PrkdcscidIl2rgtm1Wjl/SzJ (NSG) were used in the study and between 5 and 6 weeks old at the start of the experiment, weighing ~15-19g. Animals were maintained on 10-12h light cycles at ambient temperature and humidity, and fed chow and water ad libitum.
Wild animals	No wild animals were used in the study.
Reporting on sex	Animals used in studies were female due to ease of handling with NSG female mice and differences in Raji tumor progression between male and female mice.
Field-collected samples	No field collected samples were used in the study.
Ethics oversight	All animal procedures were approved by Harvard University's Institutional Animal Care and Use Committee and in compliance with National Institutes of Health guidelines.

Note that full information on the approval of the study protocol must also be provided in the manuscript.

Flow Cytometry

Plots

Confirm that:

☒ The axis labels state the marker and fluorochrome used (e.g. CD4-FITC).

☒ The axis scales are clearly visible. Include numbers along axes only for bottom left plot of group (a 'group' is an analysis of identical markers).

☒ All plots are contour plots with outliers or pseudocolor plots.

☒ A numerical value for number of cells or percentage (with statistics) is provided.

Methodology

Sample preparation	Primary human T cells, CAR-T cells, or single cell suspensions were washed several times and then stained directly with viability dye and antibodies. For blood samples, the samples were processed and washed using standard protocols to remove debris to form single cell suspensions, which were stained directly with viability dye and antibodies.
Instrument	BD LSR fortessa X-20 (5 laser setup)
Software	Flow cytometry analysis was performed in FlowJo v10.8.1
Cell population abundance	At least 10,000 relevant events were acquired for all flow cytometry analyses.
Gating strategy	In general, cells were first gated by FSC-A/SSC-A to remove debris, then gated on FSC-A/FSC-H to remove doublets, followed by live/dead exclusion. T cells were gated based on CD3 staining and lack of CD45 staining (for blood samples), followed by specific T-cell subpopulations (e.g., CD4, CD8). CAR-T cell transduction was measured using EGFR staining, while activated/inhibitory T-cell markers were measured using a combination of CD25, CD137, PD-1, and TIM-3. T-cell memory subpopulations were gated using CD45RA and CCR7. Central memory cells were defined CD45RA-CCR7+; effector memory cells, CD45RA-CCR7-, and effector cells CD45RA+CCR7-. Naive and stem cell memory cells were defined CD45RA+CCR7+.

☒ Tick this box to confirm that a figure exemplifying the gating strategy is provided in the Supplementary Information.
